# Early relapses after adjuvant chemotherapy suggests primary chemoresistance in diffuse gastric cancer

**DOI:** 10.1371/journal.pone.0183891

**Published:** 2017-09-18

**Authors:** Sharon Pattison, Catherine Mitchell, Stephen Lade, Trevor Leong, Rita A. Busuttil, Alex Boussioutas

**Affiliations:** 1 Department of Medicine, Dunedin School of Medicine, University of Otago, Dunedin, New Zealand; 2 Department of Pathology, Peter MacCallum Cancer Centre, Parkville, Victoria, Australia; 3 Sir Peter MacCallum Department of Oncology, University of Melbourne, Parkville, Victoria, Australia; 4 Department of Radiation Oncology, Peter MacCallum Cancer Centre, Parkville, Victoria, Australia; 5 Department of Pathology, University of Melbourne, Parkville, Victoria, Australia; 6 Upper Gastrointestinal Translational Research Laboratory, Peter MacCallum Cancer Centre, Parkville, Victoria, Australia; 7 Department of Medicine, University of Melbourne, Parkville, Victoria, Australia; University of North Carolina at Chapel Hill School of Medicine, UNITED STATES

## Abstract

**Background:**

Survival from gastric cancer remains poor, particularly in Western populations. Previous pre-clinical and subgroup analyses of clinical trials have suggested differing benefits from fluoropyrimidine-based chemotherapeutics for diffuse and intestinal gastric cancer. This analysis examines patterns of relapse with and without adjuvant chemotherapy after curative resection for gastric cancer in these subtypes to explore the Lauren classification as a predictive marker of benefit for fluoropyrimidine-based adjuvant chemotherapy.

**Patients and methods:**

Gastric cancer patients enrolled in an ongoing tissue banking study were analysed, 164 patients who would currently be considered for adjuvant therapy after curative resection were included in the analysis. Patients who did and did not receive adjuvant chemotherapy were compared. The primary end point was relapse free survival.

**Results:**

Approximately 50% of patients received adjuvant chemotherapy, the majority receiving a fluoropyrimidine-based regimen. The comparison of Kaplan-Meier curves for patients who did and did not receive adjuvant chemotherapy are different between patients with intestinal and diffuse gastric cancer, and suggest that there may be a benefit in intestinal gastric cancer. The hazard ratio for adjuvant chemotherapy for intestinal gastric cancer was 0.56, (95% CI 0.27–1.17), suggesting a trend towards benefit that was lacking in diffuse gastric cancer patients (1.26, 95% CI 0.70–2.38). The patterns of relapse after adjuvant chemotherapy also differed between diffuse and intestinal gastric cancer. More than 50% of diffuse gastric cancer patients who received adjuvant chemotherapy relapsed within 12 months of surgery despite similar surgical parameters.

**Conclusions:**

Lauren classification is prognostic in gastric cancer. This analysis adds further evidence that it may also be predictive of benefit for fluoropyrimidine-based chemotherapeutics, with lower chemosensitivity seen in diffuse gastric cancer. Treating diffuse and intestinal gastric cancer as separate entities, with identification of efficacious treatments for diffuse gastric cancer will help in improving outcomes from gastric cancer.

## Introduction

Survival from gastric cancer even when treated with curative intent remains poor, particularly in Western countries [1]. Epidemiological and biological evidence suggests gastric cancer is heterogeneous, with the Cancer Genome Atlas (TCGA) identifying four major molecular subtypes of gastric cancer in 2014 [2]. Despite this evidence of heterogeneity, gastric cancer is currently treated as one disease. The poor survival seen from gastric cancer is potentially influenced by lack of sensitivity to currently used chemotherapeutics in some subtypes.

The Lauren classification is a histological classification of gastric cancer first described in 1965 [3]. It divides adenocarcinoma of the stomach into two main histological subtypes, an intestinal type with gland formation, and a diffuse subtype, which demonstrates poorly cohesive malignant cells that infiltrate diffusely through the gastric wall without gland formation. In addition there is a mixed subtype that demonstrates features of both intestinal and diffuse gastric cancer, and a small proportion of cancers that cannot be classified using this system are also sometimes observed. Intestinal and diffuse gastric cancer, as well as having differing histological appearances, show differing epidemiology. Diffuse gastric cancer (DGC) occurs more frequently in younger patients, and the gender distribution is more equal, whereas intestinal gastric cancer (IGC) occurs more frequently in males and is the predominant subtype in older patients [3, 4]. The decline in incidence of gastric cancer globally is due to a decline primarily in IGC, with an increase in the incidence of DGC seen in some populations [3–6]. Lauren classification has also been identified as a prognostic variable in some cohorts, with DGC showing poorer survival [7–10]. The Lauren classification was a major clinical variable that correlated with molecular subtypes in the TCGA gastric cancer analysis, and other studies have also demonstrated molecular differences between IGC and DGC [2, 11].

Although Lauren Classification is accepted as a prognostic variable for gastric cancer, it is not recognised as being predictive of response to currently used chemotherapeutics. There is however, evidence from pre-clinical studies and subgroup analyses of clinical trials that describe DGC showing less benefit to fluoropyrimidine based chemotherapeutic regimens [12–14]. We sought to investigate the predictive value of Lauren classification for sensitivity to adjuvant fluoropyrimidine based chemotherapy by examining relapse patterns after curative surgery in intestinal and diffuse gastric cancer.

## Patients and methods

Ethical approval for the study was obtained from the Institutional Review Boards of individual hospitals involved (Peter MacCallum Cancer Centre, St Vincent’s Hospital, Royal Melbourne Hospital and Western Health, Box Hill Hospital, Cabrini Hospital). Written informed consent was obtained from study participants who were identified prior to surgery by study investigators. Overarching approval for the tissue banking cohort and this study is from the Peter MacCallum Cancer Centre Ethics Committee.

### Patients and study procedures

The Molecular Analysis of Upper Gastrointestinal Cancer (MAUGIC) cohort is a prospectively collected cohort of potentially resectable gastric cancer patients enrolled opportunistically in an on-going tissue banking study from selected hospitals in metropolitan Melbourne, Australia from 1999 to present. Clinical information was recorded at enrolment and at six monthly intervals initially. For robustness of outcome data the cohort has been linked to the Victorian Cancer Registry, a population-based cancer registry which records all cancer diagnoses in the state of Victoria, Australia. Patients with known germline *CDH1* mutations or from Hereditary Diffuse Gastric Cancer kindreds were excluded from this analysis.

Previous analyses of clinical predictors of outcome have demonstrated Lauren classification to be an independent predictor of recurrence and survival in the MAUGIC cohort [9]. The gastric cancer subset of the MAUGIC cohort, including Siewert II and III gastro-oesophageal junction (GOJ) tumours enrolled to 2009 has previously been described in more detail [9]. For the purposes of this analysis clinical outcome was censored at December 2012, and only patients who would currently routinely be considered for adjuvant (or neoadjuvant therapies) were included. Patients who would not routinely be considered for adjuvant chemotherapy (T1/T2 and node negative, incomplete surgical resection, distant metastatic disease diagnosed at surgical procedure or death prior to commencement of adjuvant treatment) were excluded. Resected tumours were reviewed by local pathologists, and tumours were staged using the American Joint Committee on Cancer (AJCC) 6^th^ edition as this was the staging manual in use during the time of enrolment of the majority of patients [15].

### Statistical considerations

The primary endpoint of this analysis was relapse free survival (RFS), measured from date of surgery to date of first documented relapse, or death from gastric cancer if recurrence was not documented. Recurrence was defined as radiological, histopathological or clinical evidence of recurrence. Self reported recurrence was confirmed with supportive medical records. The survival analysis was performed using the Survival package in R and comparisons were performed using a two-sided long-rank test [16]. Survival curves were generated using the Kaplan-Meier method.

## Results

**Fig 1:**
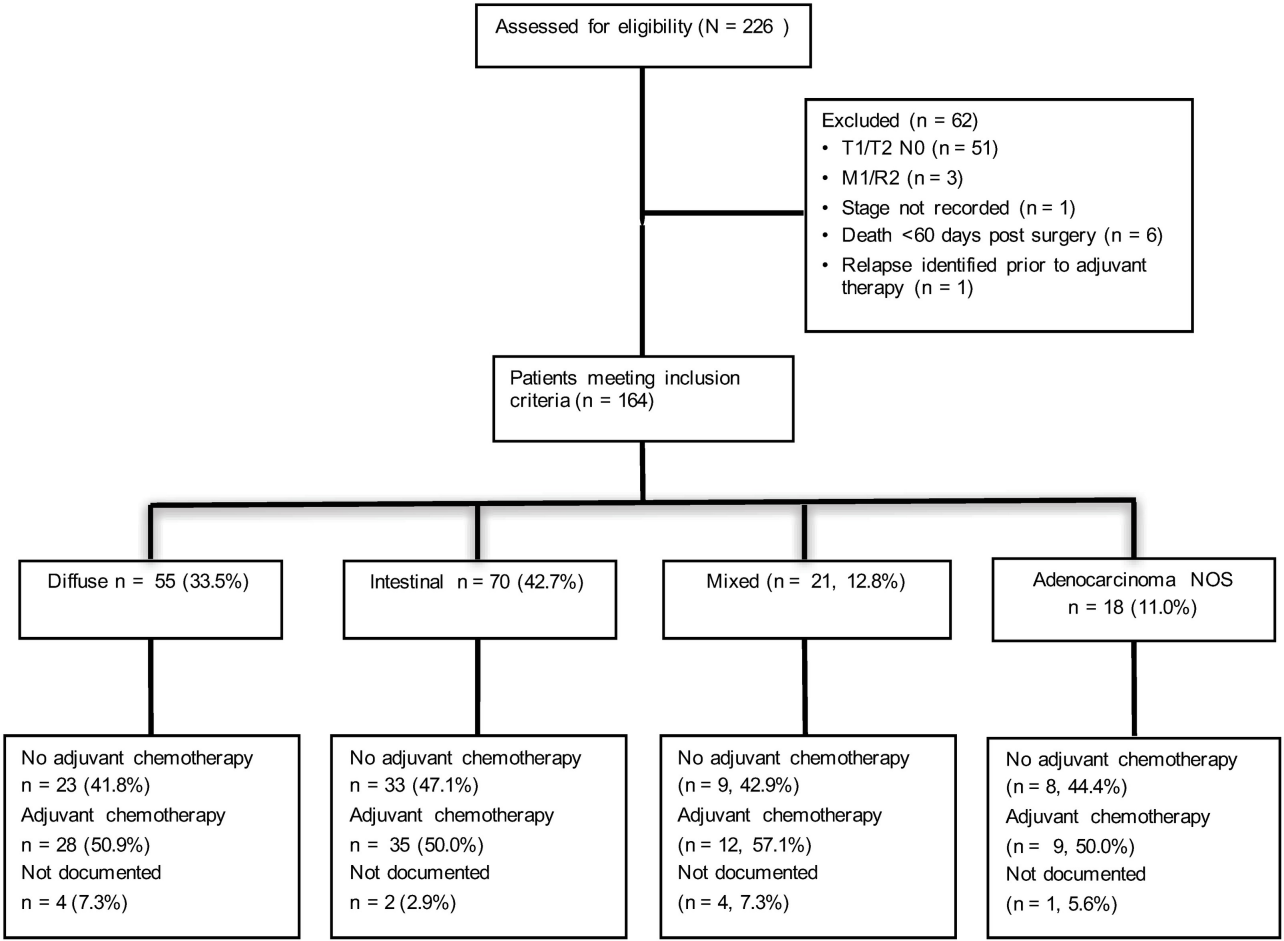
Consort diagram of patient eligibility, Lauren classification subtype and receipt of adjuvant chemotherapy.

Two hundred and twenty six patients from the MAUGIC cohort who underwent curative surgical resection were assessed for eligibility, and 164 (72.6%) met the eligibility criteria (Consort diagram, Fig 1). Fifty one (22.6%) patients were excluded due to being early stage (T1/2 N0), three (1.3%) patients had metastatic disease diagnosed at surgery or macroscopic residual disease, one (0.4%) patient did not have stage recorded, and one (0.4%) patient had relapse identified prior to commencing adjuvant chemotherapy. Six (2.7%) patients died within 60 days of surgery.

Of the 164 eligible patients 55 (33.5%) had DGC and 70 (42.7%) IGC. Of the remaining 39 patients, 21 (12.8%) had mixed gastric cancer and 18 (11.0%) adenocarcinoma not otherwise specified (NOS). The pathological stage, age, tumour location and Lauren classification subtypes are given in Table 1. The median number of lymph nodes resected for all 164 patients was 16 (range 1–56). There were no differences in age at surgery or the proportion of patients who received adjuvant chemotherapy by Lauren subtype. Compared to other Lauren subtypes intestinal tumours were more likely to be pT2 (30% vs 3.6% diffuse, 19% mixed and 16.7% adenocarcinoma NOS; Chi-squared test p = 0.03) and Stage II (51.4% vs 29.1% diffuse, 23.8% mixed and 27.8% adenocarcinoma NOS; Chi-squared test p = 0.03). Tumours that were unclassified by Lauren classification were more likely to be situated in the GOJ/cardia region (83.3%) than other subtypes. There was no difference in tumour location when DGC was compared to IGC (Chi-squared test p = 0.16). Eighty four patients (51.2%) received adjuvant chemotherapy, including approximately 50% of both DGC (n = 28) and IGC (n = 35) patients.

**Table 1:** Description of patients by Lauren classification subtype.

	Diffuse	Intestinal	Mixed	Adenocarcinoma	P value	P value
	n = 55	n = 70	n = 21	NOS n = 18	All subytpes	Diff vs Int
**Age**						
- **Median**	66	68	66	59	p = 0.17*	
- **Range**	33–86	33–87	36–85	37–81		
**T stage (AJCC 6**^**th**^ **edition)**						
- **T1**	4 (7.3%)	2 (2.9%)	0 (0.0%)	0 (0.0%)	p = 0.03	p = 0.002^¶^
- **T2**	2 (3.6%)	21 (30.0%)	4 (19.0%)	3 (16.7%)		
- **T3**	47 (85.5%)	46 (65.7%)	17 (81.0%)	15 (83.3%)		
- **T4**	2 (3.6%)	1 (1.4%)	0 (0.0%)	0 (0.0%)		
**N stage (AJCC 6**^**th**^ **edition)**						
- **N0**	16 (29.1%)	17 (24.3%)	2 (9.5%)	2 (11.1%)	p = 0.03	p = 0.10^¶^
- **N1**	20 (36.4%)	40 (57.1%)	9 (42.9%)	14 (77.8%)		
- **N2**	14 (25.5%)	10 (14.3%)	7 (33.3%)	2 (11.1%)		
- **N3**	5 (9.1%)	3 (4.3%)	3 (14.3%)	0 (0.0%0		
**AJCC stage (6**^**th**^ **edition)**						
- **Ib**	4 (7.3%)	1 (1.4%)	0 (0.0%)	0 (0.0%)	p = 0.03^¶^	p = 0.03^¶^
- **II**	16 (29.1%)	36 (51.4%)	5 (23.8%)	5 (27.8%)		
- **III**	29 (52.7%)	30 (42.9%)	13 (61.9%)	13 (72.2%)		
- **IV**	6 (10.3%)	3 (4.3%)	3 (14.3%)	0 (0.0%)		
**Tumour location**						
- **GOJ/Cardia**	13 (23.6%)	20 (28.6%)	2 (9.5%)	15 (83.3%)	p < 0.001^¶^	p = 0.16^¶^
- **Body**	34 (61.8%)	32 (45.7%)	15 (71.4%)	2 (11.1%)		
- **Antrum/ pylorus**	8 (14.5%)	18 (25.7%)	4 (19.0%)	1 (5.6%)		
**Adjuvant chemotherapy**						
- **No**	23 (41.8%)	33 (47.1%)	9 (42.9%)	8 (44.4%)	p = 0.83^¶^	
- **Yes**	28 (50.9%)	35 (50.0%)	12 (57.1%)	9 (50.0%)		
- **Not documented**	4 (7.3%)	2 (2.9%)	0 (0.0%)	1 (5.6%)		

NOS–not otherwise specified; GOJ–gastro-esophageal junction; Diff–Diffuse; Int–Intestinal.

*Kruskal-Wallis test.

^¶^Chi-squared test.

The description of pathological stage, age, tumour location and Lauren subtype by receipt of adjuvant chemotherapy is provided in Table 2. Patients who did not receive adjuvant chemotherapy where older than those who did (adjuvant chemotherapy median age 61.5 years, no adjuvant chemotherapy median age 71 years, Kruskal Wallis test p <0.001), and of lower stage (54.8% stage Ib/II no adjuvant chemotherapy, 26.2% stage Ib/II adjuvant chemotherapy, Chi-squared test p = 0.002). There were no differences in tumour location or Lauren classification between patients who did and did not receive adjuvant chemotherapy.

**Table 2:** Description of patients by receipt of adjuvant chemotherapy.

	No adjuvant chemotherapy n = 73	Adjuvant chemotherapy n = 84	P value
**Age**			
** - Median**	71.0	61.5	p < 0.001*
** - Range**	38–87	33–82	
**AJCC stage**			
** - Ib**	3 (4.1%)	1 (1.2%)	p = 0.002^¶^
** - II**	37 (50.7%)	21 (25.0%)	
** - III**	31 (42.5%)	52 (61.9%)	
** - IV**	2 (2.7%)	10 (11.9%)	
**Tumour location**			
** - GOJ/ Cardia**	23 (31.5%)	24 (28.6%)	p = 0.72^¶^
** - Body**	38 (52.1%)	42 (50.0%)	
** - Antrum/ Pylorus**	12 (16.4%)	18 (21.4%)	
**Lauren classification**			
** - Diffuse**	23 (31.5%)	28 (33.3%)	p = 0.97^¶^
** - Intestinal**	33 (45.2%)	35 (41.7%)	
** - Mixed**	9 (12.3%)	12 (14.3%)	
** - Adenocarcinoma NOS**	8 (11.0%)	9 (10.7%)	

GOJ–gastro-esophageal junction; NOS–not otherwise specified.

*Kruskal-Wallis test.

^¶^Chi-squared test.

Information on chemotherapy regimen was available for 95% (n = 80) of patients receiving adjuvant chemotherapy, all of whom received a fluoropyrimidine (5-fluorouracil or capecitabine) based regimen, 49% as a doublet (n = 2, 2.4% platinum + fluoropyrimidine) or triplet combination (n = 39, 46.4%, anthracycline + platinum + fluoropyrimidine). For four patients (4.8%) the chemotherapy regimen administered was unknown. Thirteen percent (n = 22) of all patients received neoadjuvant chemotherapy or chemoradiotherapy. Ten of these patients also received adjuvant chemotherapy (four receiving neoadjuvant and adjuvant chemotherapy, five receiving neoadjuvant chemotherapy and post-operative chemoradiotherapy and one receiving neoadjuvant chemoradiotherapy followed by adjuvant chemotherapy), and 12 did not receive adjuvant therapy (eight patients received neoadjuvant chemoradiotherapy, and four neoadjuvant chemotherapy alone). Forty five percent (n = 74) of patients underwent adjuvant chemoradiotherapy. One patient did not receive chemotherapy with radiation in the adjuvant setting for reasons unknown.

**Fig 2:**
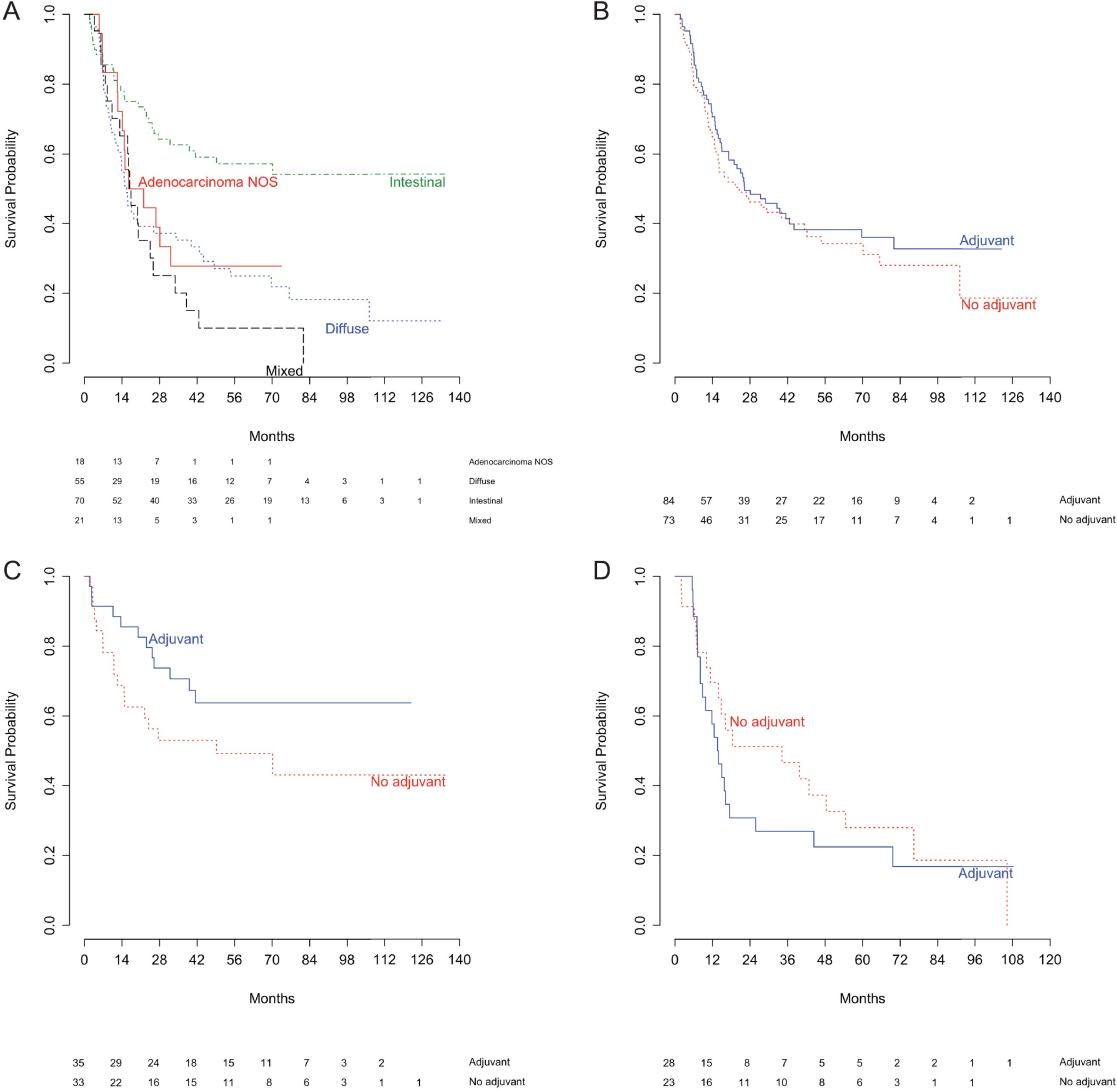
Kaplan-Meier estimates of relapse free survival among patients eligible for adjuvant therapy after curative resection of gastric cancer. (A) By Lauren classification subtype. (B) By receipt of adjuvant chemotherapy in all patients. (C) By receipt of adjuvant chemotherapy in patients with intestinal gastric cancer only. (D) By receipt of adjuvant chemotherapy in patients with diffuse gastric cancer only.

Median follow up duration for all eligible patients was 27.8 months (range 2–135), median follow up duration in patients without relapse was 63 months (range 2–135). The number of relapses, median time to relapse, and RFS are described in Table 3. As in the published description of the surgically resectable MAUGIC cohort, patients with IGC showed the best RFS of all Lauren classification subgroups (Fig 2A, Table 3, p <0.0001) [9]. Consistent with this there was a smaller proportion of relapses seen in IGC patients (Table 3). The survival curves for all patients irrespective of whether they received adjuvant chemotherapy are nearly identical (Fig 2B) reflecting the median RFS of 25.9 months in patients who received adjuvant chemotherapy and 24.0 months in those who did not (Table 3). In DGC patients, median RFS in those with adjuvant chemotherapy was 13.7 months, and without was 34.2 months. In patients with IGC who received adjuvant chemotherapy, median RFS was not reached, and was 49.2 months in patients who did not receive adjuvant chemotherapy. Although a strong trend was observed none of these comparisons reached statistical significance (Table 3). Examination of the Kaplan-Meier curves, hazard ratio (HR) and proportion of patients relapsing with IGC suggest some benefit from adjuvant chemotherapy with separation of the curves, the HR for adjuvant chemotherapy being below one (HR 0.56, 95% CI 0.27–1.17, p = 0.12, Fig 2C) and the smallest proportion of patients relapsing (34.3%, Table 3). In contrast, in DGC patients the separation of the curves is much less apparent, and no difference was observed in the proportion of patients relapsing with and without adjuvant treatment suggestive of lack of benefit (HR 1.26, 95% CI 0.70–2.38, p = 0.47, Fig 2D, Table 3).

**Table 3:** Relapse free survival by receipt of adjuvant chemotherapy.

	N	Relapses (% parent group)	Median RFS (months)	95% CI (months)	Hazard ratio	95% CI	Log rank test p value
Lauren classification							
- Intestinal	70	29 (41.4%)	Not reached	41.4-not reached	1	-	<0.0001
- Diffuse	55	42 (76.4%)	15.7	12.5–39.8	2.58	1.60–4.15	
- Mixed	21	19 (90.5%)	17.3	13.2–33.8	3.36	1.87–6.04	
- Adenocarcinoma NOS	18	13 (72.2%)	19.3	14.1-not reached	2.44	1.26–4.74	
All patients*							
- No adjuvant chemo	73	48 (65.8%)	24.0	16.1–49.2	1	-	0.45
- Adjuvant chemo	84	51 (60.7%)	25.9	20.0–69.7	0.86	0.58–1.28	
Diffuse*							
- No adjuvant chemo	23	18 (78.3%)	34.2	13.8-not reached	1	-	0.47
- Adjuvant chemo	28	21 (75.0%)	13.7	8.81–44.4	1.26	0.70–2.38	
Intestinal*							
- No adjuvant chemo	33	17 (51.5%)	49.2	15.0-not reached	1	-	0.12
- Adjuvant chemo	35	12 (34.3%)	NA	41.4-not reached	0.56	0.27–1.17	

RFS–relapse free survival; CI–confidence interval; NOS–not otherwise specified.

*Excluding patients for whom receipt of adjuvant chemotherapy is not documented.

Sixty one percent (n = 51) of patients who received adjuvant chemotherapy relapsed, with median time to relapse of 15 months (range 2–44 months). In DGC, 75% (n = 21) of patients receiving adjuvant chemotherapy relapsed (median time to relapse 10 months, range 6–44 months) and in IGC patients 34% (n = 12) relapsed (median time to relapse 23 months, range 2–41 months).

**Fig 3:**
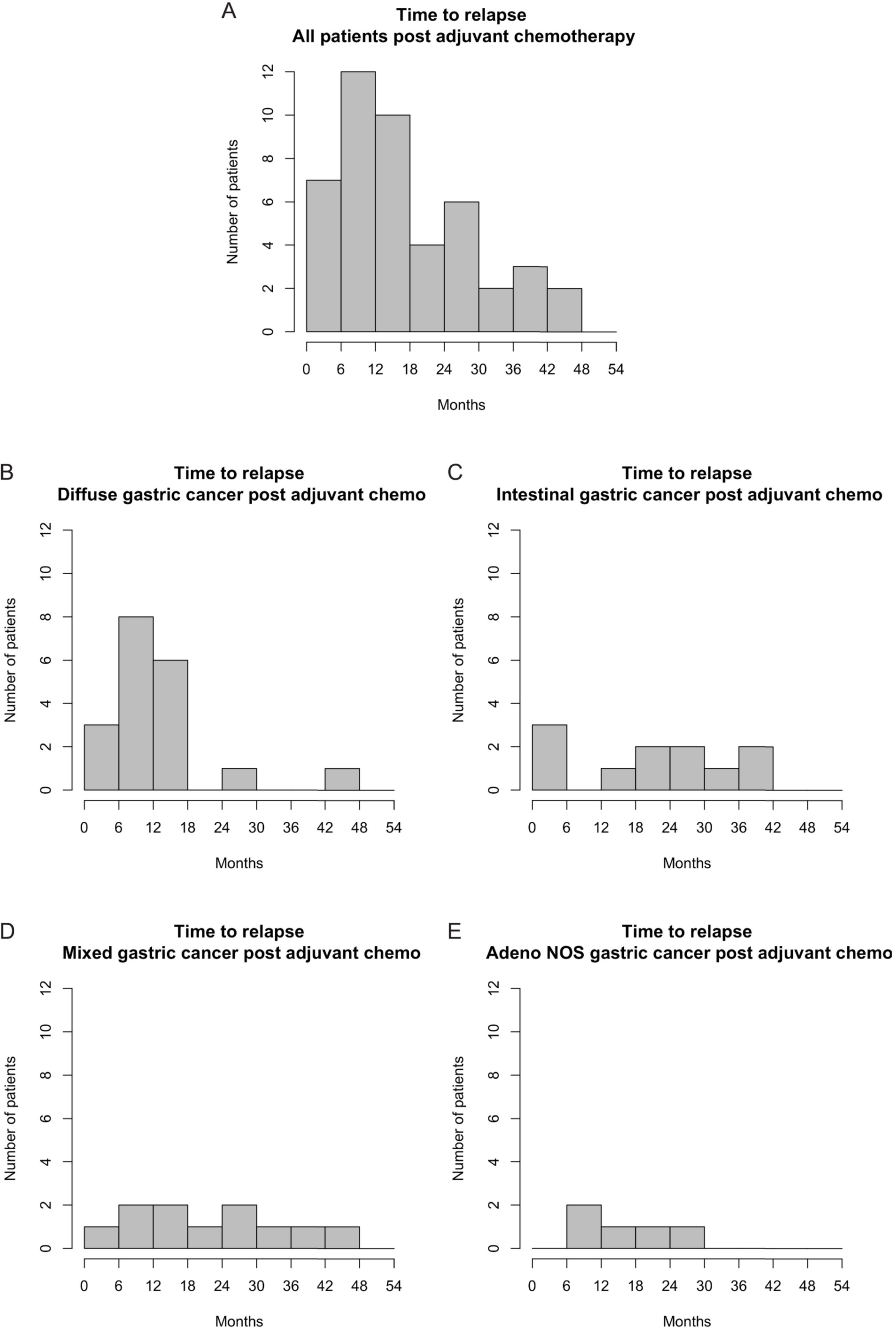
Histograms depicting time to relapse in months in patients who received adjuvant chemotherapy. (A) All patients. (B) Diffuse gastric cancer patients only. (C) Intestinal gastric cancer patients only. (D) Mixed gastric cancer patients only. (E) Patients with adenocarcinoma not otherwise specified only.

The histograms in Fig 3 show the different patterns of relapse for those patients that received adjuvant chemotherapy by Lauren subtype. Most recurrences occurred within 18 months of surgery (Fig 3A), and further analyses based on Lauren subtype revealed that this was almost entirely as a result of DGC patients (Fig 3B). More than half of the relapses seen in DGC patients occurred within the first 12 months after surgery, despite similar clinical characteristics. By comparison, there is a steady state of relapse in IGC, mixed GC and patients with tumours not classifiable (Fig 3C–3E). A similar recurrence pattern was seen in patients who did not receive adjuvant chemotherapy, with the majority of patients relapsing within 18 months of surgery, however this peak appears to be contributed to by all Lauren subtypes in this setting (S1 Fig).

## Discussion

There is strong evidence that IGC and DGC are distinct entities with epidemiological, prognostic and molecular differences in addition to their different histologic morphology [2, 4, 9, 11]. This analysis provides further evidence that Lauren classification is predictive of response to fluoropyrimidine-based chemotherapeutics. Distinct patterns are seen in RFS with and without adjuvant chemotherapy when DGC and IGC are examined separately. In IGC, the Kaplan-Meier analysis suggests some benefit from adjuvant fluoropyrimidine-based chemotherapy with the curve for adjuvant chemotherapy above that for no adjuvant chemotherapy for the duration of follow up (Fig 2C), with a HR less than 1. In DGC, the Kaplan-Meier curves are reversed, with the curve for patients receiving adjuvant chemotherapy sitting below patients recieving no adjuvant chemotherapy (Fig 2D), and a HR of greater than 1. The histograms of time to relapse after adjuvant chemotherapy show a distinct pattern in DGC (Fig 3). For other Lauren subtypes there is a slow, steady rate of relapse, in contrast in DGC the majority of relapses occur within the first 18 months, with more than half in the first 12 months from surgery suggesting minimal benefit, if any, from the addition of adjuvant chemotherapy.

The MAUGIC cohort is a unique cohort of gastric cancer patients of predominantly European ethnicity [9]. The enrolment period of the MAUGIC cohort used in this analysis, 1999–2009, encompasses a time when treatment of gastric cancer was changing, with the publication of the Intergroup 0116 study in 2001 and the MAGIC study in 2006, being the seminal studies showing improvement in survival in Western gastric cancer populations with adjuvant chemoradiotherapy and peri-operative chemotherapy respectively [17, 18]. The small numbers of patients who received neoadjuvant (n = 22, 13%) and adjuvant (n = 84, 51%) therapies reflects this enrolment period. This enrolment period provides an opportunity to examine the effects on survival from the different treatment modalities. The observational nature of this analysis is one of its limitations, as treatments were not randomised, but determined by the treating physicians. In addition, age and stage, variables that are also prognostic for gastric cancer, both impact whether a patient is offered adjunctive treatments to surgery, and therefore are likely to confound any survival differences seen due to additional therapies. In this sub analysis of the MAUGIC cohort, patients who did not receive adjuvant chemotherapy were older and of lower stage, variables associated with poorer survival and improved survival respectively in gastric cancer [7–9]. The size of the MAUGIC cohort limits the ability to tease out the impact of these confounding variables. In the analysis of the complete resectable MAUGIC cohort there was no interaction seen between Lauren classification, age at surgery or stage, suggesting that the relapse patterns seen in the adjuvant chemotherapy eligible patients are not likely to be strongly influenced by stage or age [9]. The majority of patients in the MAUGIC cohort were Eastern Co-operative Oncology Group performance status 0 or 1 suggesting that fitness pre-surgery for the majority of patients would not have precluded adjuvant therapy, however a full assessment of co-morbidities before and after surgery is not available for the cohort and the impact of these can therefore not be taken into account [9].

There is existing evidence that support the hypothesis that DGC and IGC may have differential responses to currently used chemotherapeutics. A pre-clinical study which identified two intrinsic genomic subtypes of gastric cancer, G-INT and G-DIF, with 64% concordance to the respective Lauren classification subtypes, found G-INT cell lines were more sensitive to 5-FU and oxaliplatin and more resistant to cisplatin than G-DIF cell lines [14]. A retrospective analysis from a single institute in Italy of 248 patients which investigated response to chemotherapy in metastatic gastric cancer found response rate (RR) was lower in patients with DGC (20.4%) when compared to proximal non-diffuse gastric cancer (46.1%) and distal non-diffuse gastric cancer (30.3%) [19]. Subgroup analysis of the JCOG9912 trial, investigating single agent 5-fluoruracil (5-FU) compared to S-1 or the combination of cisplatin and irinotecan chemotherapy in metastatic gastric cancer found a benefit for the cisplatin/irinotecan combination over 5-FU in patients with DGC but not IGC [12]. Retrospective subgroup analysis of the Intergroup 0116 trial revealed patients with DGC did not appear to benefit from adjuvant 5-FU based chemoradiotherapy, with a median overall survival (OS) from surgery alone of 42 months, and 31 months with the addition of chemoradiotherapy [13].

Relapse patterns after adjuvant radiotherapy were not specifically examined in this analysis. The majority of patients who received adjuvant chemotherapy received concurrent chemoradiotherapy (n = 73, 87%), providing indirect evidence that there is a differential benefit from radiotherapy in DGC and IGC. This is also seen in the subgroup analysis of the ARTIST trial, which examined the addition of radiotherapy to capecitabine and cisplatin chemotherapy, and also demonstrated a lack of benefit for the addition of radiotherapy in DGC [20].

These results are contrasted however by the recently published retrospective analysis examining markers of recurrence and survival in the MAGIC trial cohort where Lauren subtype was not identified as being associated with pathological response to chemotherapy or survival [21]. However, only 15% of patients receiving neo-adjuvant chemotherapy (24 patients) were DGC and, as discussed by Smyth et al, this analysis is likely underpowered to evaluate this subset.

The TCGA gastric cancer cohort identified four major subtypes of gastric cancer and the Lauren subtype of DGC was significantly enriched in the genomically stable subgroup [2]. IGC was found in three distinctly different molecular subgroups: Epstein Barr virus (EBV), microsatellite instability (MSI) and chromosomal instability [2]. Cristescu et al also identified four major subtypes of gastric cancer, with the majority of the mesenchymal subtype being DGC and showing poorer prognosis than their other identified subtypes [22]. The MSI subtype was enriched for IGC and showed improved survival [22]. In DGC, the proposed molecular drivers, for example gain of function *RHOA* mutations, structural re-arrangements involving *ARHGAP26* (a GTPase involved in RHO signalling) and loss of *CDH1* have been reported to occur in a mutually exclusive manner [2, 23]. These, and other studies, suggest unique molecular backgrounds for DGC and IGC which likely contribute to variability in survival, response to currently used treatments and response to investigational agents in clinical trials. Consistent with an impact of genomic background on response to treatment, inhibition of RhoA in DGC cell lines and xenografts has been shown to increase sensitivity to cisplatin [24]. Gain of function mutations in the GTPase *RHOA* are one of the recurrent mutational events identified in DGC but not IGC, and increased RhoA activity has been correlated with poorer overall survival in DGC, but again not in IGC [2, 23–25].

This analysis adds to the growing evidence of a potential predictive role for the Lauren classification with regards to fluoropyrimidine-based chemotherapy, in addition to its prognostic significance. Survival from gastric cancer in Western populations remains poor, and while awaiting molecular markers to guide individualised patient treatment, investigation and treatment of diffuse and intestinal gastric cancer as separate entities will improve our ability to tailor treatments to individual patients.

## Supporting information

S1 Fig Histograms depicting time tor relapse in months in patients who did not receive adjuvant chemotherapy. (A) All patients. (B) Diffuse gastric cancer patients only. (C) Intestinal gastric cancer patients only. (D) Mixed gastric cancer patients only. (E) Patients with adenocarcinoma not otherwise specified only.
(TIF)

S1 Table Dataset. (XLSX)
