# Supplementary figures and images for: Early relapses after adjuvant chemotherapy suggests primary chemoresistance in diffuse gastric cancer
Source: PLoS One. 2017 Sep 18;12(9):e0183891. doi: 10.1371/journal.pone.0183891 (PMC5602536; doi:10.1371/journal.pone.0183891)

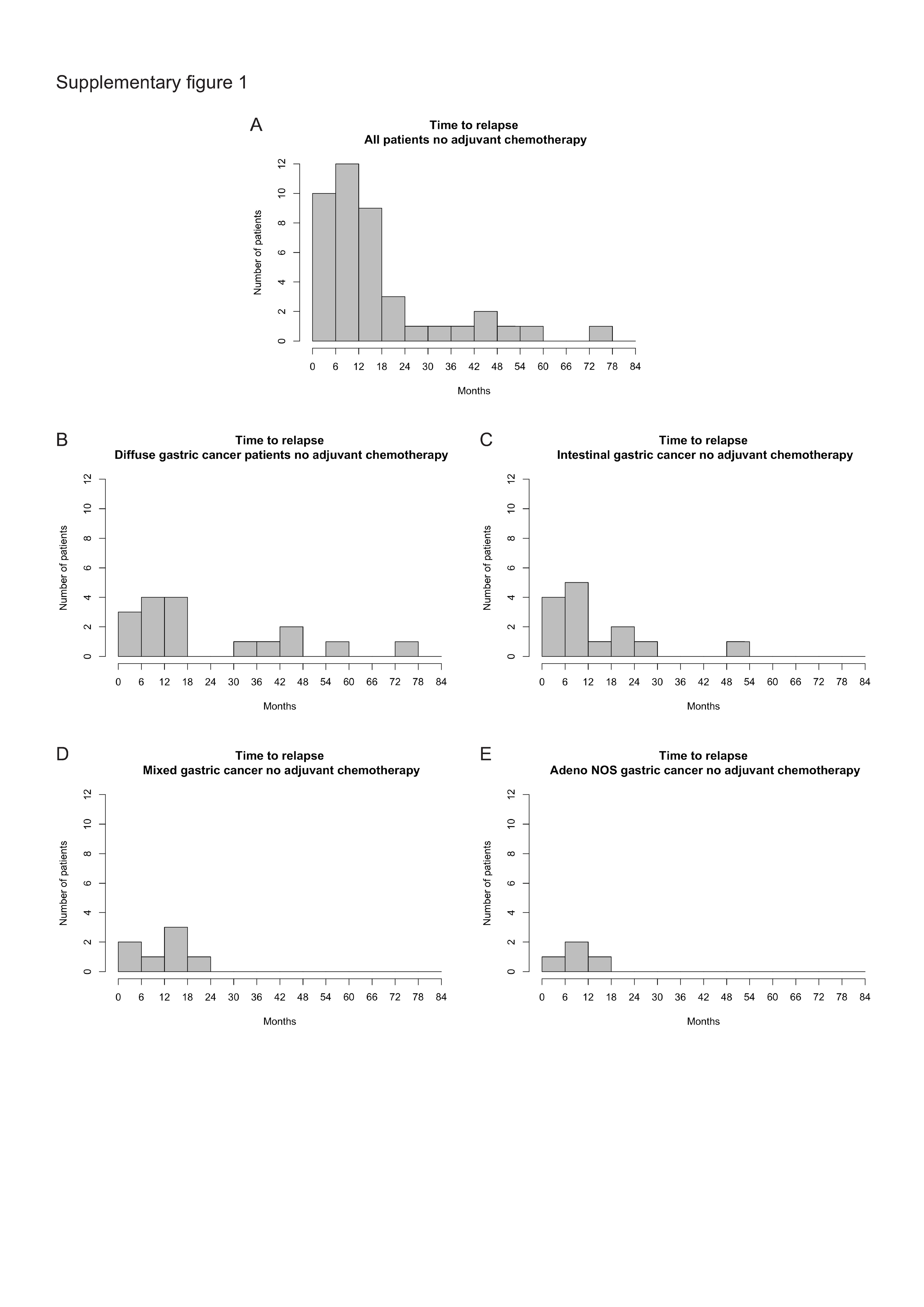

Supplement: S1 Fig — (A) All patients. (B) Diffuse gastric cancer patients only. (C) Intestinal gastric cancer patients only. (D) Mixed gastric cancer patients only. (E) Patients with adenocarcinoma not otherwise specified only. (TIF) [file pone.0183891.s001.tif]
